# Effects of periodontitis on the development of asthma: The role of photodynamic therapy

**DOI:** 10.1371/journal.pone.0187945

**Published:** 2017-11-16

**Authors:** Larissa Carbonera Candeo, Nicole Cristine Rigonato-Oliveira, Aurileia Aparecida Brito, Rodrigo Labat Marcos, Cristiane Miranda França, Kristianne Porta Santos Fernandes, Raquel Agnelli Mesquita-Ferrari, Sandra Kalil Bussadori, Rodolfo Paula Vieira, Adriana Lino-dos-Santos-Franco, Ana Paula Ligeiro-Oliveira, Anna Carolina Ratto Tempestini Horliana

**Affiliations:** 1 Post Graduate Program in Biophotonics Applied to Health Sciences, University Nove de Julho (UNINOVE), São Paulo, Brazil; 2 Division of Biomaterials and Biomechanics, Department of Restorative Dentistry, OHSU School of Dentistry, Portland, Oregon, United States of America; 3 Post Graduate Program in Rehabilitation Sciences, Universidade Nove de Julho, UNINOVE, São Paulo, Brazil; 4 Instituto Brasileiro Ensino/Pesquisa em Imunologia Pulmonar e do Exercício, São Jose dos Campos, São Paulo, Brazil; Massachusetts General Hospital, UNITED STATES

## Abstract

To evaluate whether periodontitis modulates lung inflammation in an experimental model of asthma as well as the photodynamic therapy (PDT) is associated with a reduction of lung inflammation. Seventy-two BALB/c male mice (~2 months) were randomly divided into 8 groups (n = 9): Basal, Periodontitis (P), P+PT, P+PT+PDT, Asthma (A), A+P, A+P+PT, and A+P+PT+PDT. Periodontitis was induced by using the ligature technique and asthma was induced by ovalbumin (OVA). PT was performed with curettes and PDT with methylene blue (0.005%), λ = 660nm, with a radiant exposure of 318J/cm^2^. After 43 days, euthanasia was carried out prior to lung and mandible morphological analyzes. All of the manipulations of the animals were performed by only one operator. The total and differential cell counts and cytokines IL-4, IL-5, IL-10, IFN-γ, TNF-α, IL-1β, and IL-6 were evaluated in the bronchoalveolar lavage (BAL) and in the serum. Mucus and alkaline phosphatase were also quantified. Statistical analyzes were performed by a blinded statistician. One-way analysis of variance (ANOVA) was employed, followed by the Student-Newman-Keuls test. Periodontitis group (P) increased alkaline phosphatase and bone resorption (p<0.05), validating the experimental model of periodontitis. The A group and the P group increased the total amount of cells (p <0.05) in the BAL. However, in the A+P group, there was a decrease in these cells, except for in the A+P+PT+PDT group (p<0.05). The asthma group increased the Th2 cytokines and P group increased the Th1 cytokine profile, and A+P+PT+PDT group increased IL-10 cytokine. Mucus was increased for the A and P groups. In conclusion, periodontitis in the asthmatic mice reduced the inflammatory migrated cells in the BAL (eosinophils, lymphocytes, macrophages). In addition, it reduced the levels of the IL-4 and TNF-α cytokines, which was also accompanied by a decreased mucus production. After PDT treatment the total cell count increased however, this increase was not accompanied by a pro-inflammatory cytokines release. Only in PDT group the anti-inflammatory IL-10 was increased. Further studies are needed to understand this mechanism of action.

## Introduction

Periodontal disease is an infectious disease of the supporting structures of teeth that affects over 47% of American adults [1]. In the elderly, 65 and older, the prevalence rate increases to over 70%. The cost that is associated with the treatment of PD represents a significant fraction of all of the expenses related to dental care, which totals on average, $113 billion a year in the US [2]. Periodontitis is characterized by an acute inflammatory process, osteoclast activity, as well as connective tissue destruction [3,4], which is then characterized by a pro-inflammatory profile of cytokine release, known as Th1 [3]. Among the cytokines with a Th1 profile, we can cite IL-1β, IL-6 and TNF-α [5–8]. Periodontal disease is often associated with other chronic systemic conditions, such as cardiovascular diseases [9], diabetes [10], and asthma. [11].

Asthma is defined as a chronic inflammation of the airways, with recurrent and reversible episodes of dyspnea, chest stiffness, coughing and wheezing [12]. Its prevalence can vary from 1% to 18%, depending upon the studied population [13]. According to the World Health Organization (WHO), it estimates that 235 million people worldwide suffer from asthma [14]. The socio-economic implications are considerable, when one considers work absenteeism, hospitalization costs, medicines, a decrement in the quality of life, and premature death [13]. The respiratory system becomes hyperresponsive and [15] the major consequence is a reversible mechanical obstruction of the airways [16]. Although the primary cause may vary, [15] it can be triggered by several factors, classified as predisposing, causal and contributory [17]. After the sensitization phase, the asthmatic patient presents with eosinophil infiltrate, activated mast cells on the airways surface, together with activated T lymphocytes, with a profile of cytokine release, known as Th2. Among the cytokines with a Th2 profile, we can highlight IL-4, IL-5 and IFN-γ, due their effects during the allergic responses [18,19]. In addition, the Th2 profile has also been described as being involved in the progression of periodontitis [3].

Some studies linking periodontitis and asthma have been proposed [19–28]. The lack of standardization for a periodontitis diagnosis and the inclusion of patients with different ages [19] make it difficult to compare studies. As for asthma, a diagnosis is often made by a self-reporting of the disease [19]. Further studies are needed in order to elucidate the link between these two pathologies [19–29]. In a recent study with 5.976 patients, a positive association was found between periodontitis and asthma [11], while there is an inverse association when the patients have been taking antiasthmatic medication [11]. The causal relationship between them is still unclear [30]. Allergies have been negatively associated with clinical attachment loss [22] hypothesizing that a periodontopathogenic colonization of an oral cavity could have a protective effect on an allergic disease [24, 19]. Other authors [20] have found an inverse association between a clinical attachment loss and asthma. Corroborating with human studies, some authors [8] evaluated the immunoregulatory mechanism of asthma during a periodontitis. This study determined if a subcutaneous infection with *Porphyromonas gingivalis* exerted a regulatory effect on the allergic airway inflammation. The authors showed a reduction of lung inflammatory cells, as well as cytokines, after a *P*. *gingivalis* infection, prior to an allergen sensitization with ovalbumin (OVA).

The standard PD treatment aims to reduce the biofilm microorganisms through scaling and root planning, followed by an oral hygiene control by the patient. However, it is not able to eliminate the subgingival pathogens and the calculus [31]. There may be a bacterial re-colonization at inaccessible periodontal sites. *Aggregatibacter actinomycetemcomitans* and *Porphyromonas gingivalis* have been found in infected pulmonary fluids [32]. Nowadays, antibiotics are indicated for a restricted group of patients, as an adjuvant to a periodontal treatment [33], due to the risk of a bacterial resistance development [34]. Antimicrobial photodynamic therapy (PDT) is an adjuvant to a periodontal treatment, in order to reduce the amount of microorganisms in the localized infections of the subgingival sites [35]. The principal advantage of this adjuvant is that the light and the photosensitizer reach places where the conventional treatment with curettes only has partial access. The mechanism is based upon the activation of a photosensitizer drug, by light at a suitable wavelength, generating oxidative species, such as hydroxyl radicals, superoxide and singlet oxygen [36]. These species act in the bacteria organelles, damaging structures and loosing essential functions for survival [37]. However, there is an absence of literature reports regarding a bacterial resistance to PDT [38,39]. Cationic photosensitizers bind to bacteria due to electrostatic interactions, since there is a negative potential in the cell’s surface [39]. Regarding this concept, phenothiazinium dyes have been widely studied as photosensitizers [40–43], with methylene blue and toluidine blue being the main ones. Methylene blue presents a broad spectrum of activity against bacteria, being effective in the inactivation of *P*. *gingivalis*, *P*. *intermedia*, and *A*. *actinomycetemcomitans* bacteria [43–46], with a key role in a periodontitis. Despite the advantages of PDT, there is a lack of well-designed clinical studies for a proper evaluation of this therapy [47]. There are no reports of a bacterial resistance or side effects, with a preservation of the oral microbiota and a low toxicity, [43] unlike a treatment with antibiotics or a mouthwash with chlorhexidine. Notwithstanding the advantages of PDT, well-designed studies on this subject are needed [48–51].

There is an important gap in the literature, evaluating whether a periodontitis is capable of influencing the development of a pulmonary disease. In addition, if the suppression of the infectious agent of the periodontium is able to influence the inflammatory parameters of asthma. Therefore, the hypothesis of this study has been to evaluate whether periodontitis modulates lung inflammation in an experimental model of asthma as well as the photodynamic therapy (PDT) is associated with a reduction of lung inflammation

## Material and methods

**Fig 1:**
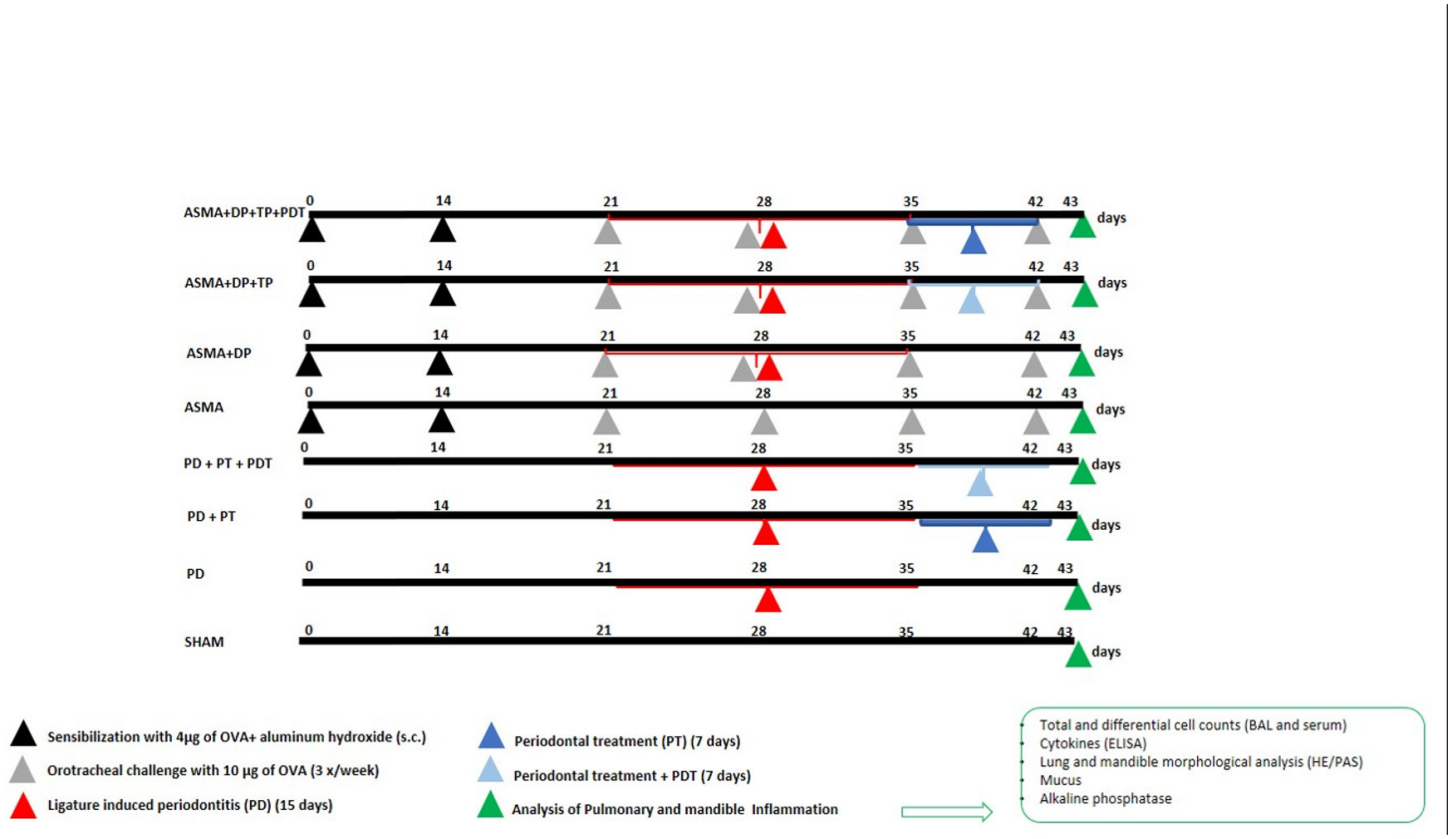
Experiment timeline and design.

This study was approved by the Animal Ethics Committee of the University Nove de Julho (UNINOVE), São Paulo, Brazil, under #020/2015. The mice were maintained in a constant temperature of 22°C to 25°C, with a 12-h light/dark photoperiod, under artificially controlled ventilation, with a relative humidity ranging from 50% to 60%. Rations (NUTRILAB CR-1^®^) and water were provided *ad libitum*. Seventy-two BALB/c male mice (~2 months) were randomly divided into 8 groups (n = 9): 1) Basal—without any induction of a disease, 2) P—Induction of Periodontitis, 3) P+TP—Induction of Periodontitis + Standard Periodontal Treatment, 4) P+PT+PDT—Induction of Periodontitis + Standard Periodontal Treatment + Photodynamic Therapy, 5) Asthma—Induction of Asthma, 6) Asthma +P—Induction of Asthma + Induction of Periodontitis, 7) Asthma+P+TP—Induction of Asthma + Induction of Periodontitis + Standard Periodontal Treatment, 8) Asthma+P+TP+PDT—Asthma Induction + Induction of Periodontitis + Standard Periodontal Treatment + Photodynamic Therapy. All of the manipulations of the animals (periodontitis induction, periodontal treatment, PDT) were performed by only one operator (Candeo LC). Seventy-two animals were identified. The randomization of the animals was performed (Microsoft Excel, Version 2013) by separating them into 8 blocks (groups) of 9 animals. For the induction of asthma, the animals were injected subcutaneously with 4 μg ovalbumin (OVA) (SIGMA^™^), together with aluminum hydroxide solution, on the first day of the experiment (sensitization), and then 14 days thereafter (booster). From the 14^th^ day, the animals were submitted to a nebulization (challenge) with 10 μg OVA, 3 times a week, for 2 weeks [52–55] (Fig 1).

Periodontitis was induced by using a modification of a previously described method of ligature-induced periodontitis in mice [56–58]. The ligature method is detailed at S1 Fig. The method of the periodontitis induction was realized by one operator when using an ophthalmological nylon silk ligature of 6–0 (SHALON^®^, São Paulo, Brazil). It was gently introduced into the interproximal area between the first mandibular molar and the second mandibular molar with two curved needle-holders (DLMICOF^®^, Sao Paulo, Brazil) that were developed for this study. The silk ligature was gently tied in order to avoid damaging the periodontal area around the first molar. In order to perform these procedures, the mice were anesthetized with an intraperitoneal injection of ketamine (100 mg/kg) (DOPALEN^®^, VETBRANDS, São Paulo, Brazil) and the muscle relaxant xylazine (10 g/kg) (ANASEDAN^®^, VETBRANDS, Brazil). They remained sedated for approximately 90 minutes. After 15 days, the ligatures were removed and the periodontal treatments were performed in a standardized manner in the groups P+PT, P+PT+PDT, Asthma+P+PT, and Asthma+P+PT+PDT (Fig 1). No antibiotics or anti-inflammatories were administered. A Mini Five 5/6 curette was used and crown-root scrapings/ planning were performed on the vestibular lingual, mesial and distal faces. PDT was then employed as an adjuvant therapy to the periodontal treatments in the P+PT+PDT and Asthma+P+TP+PDT groups. For such, the photosensitizer methylene blue (0.005%—CHIMIOLUX, DMC, São Paulo, Brazil) was administered with a syringe, cartridge and needle (with a stop and without a bevel) at the two sites (vestibular and lingual). After three minutes, the periodontal pockets were irradiated with a red laser (λ = 660 nm ±10nm) (THERAPY XT, DMC, São Carlos, São Paulo, Brazil—ANVISA 80030810157). The radiant power of the appliance is 100 mW. The spot (area) was 0.02827 cm^2^. The radiant energy delivered per point was 9J in 90 seconds. The radiant exposure was 318 J/cm^2^ and irradiance was 3.5W/cm^2^. In the vestibular face 9J were applied and another point in the lingual face (9J) of the right first molar also applied [59], (Table 1). followed by abundant rinsing with water for complete removal of the methylene blue. We irradiated with a small spot (0,02827mm^2^) because the tooth size of the 1^st^ mandibular molar of mice is about 1.44 ± 0.011 mm at vestibular face and 0.80 ± 0.019 mm in the buccolingual length. [60]. All periodontal treatment (with or without PDT) was performed in a single session Euthanasia and posterior analyzes of the material were performed, 43 days after the beginning of the study The complete timeline of the induced ligature (15 days), the periodontal treatment (7 days), the PDT (7 days) and the euthanasia (43 days), for the 8 groups of this study, is detailed in Fig 1. After the experimental period, the mice were euthanized with an overdose of ketamine (1.6 g/10 ml of solution) and xylazine (3g/100ml of solution).

**Table 1:** Parameters of red laser.

Parameter	Red laser
Wavelength	660 nm
Radiant power	100 mW
Exposure duration	90s
Beam spot size at target	0.02827 cm^2^
Radiant energy	9J
Irradiance	3.5 W/cm^2^
Radiant exposure	318 J/cm^2^
Anatomical location	Vestibular and palatal surface
Number of irradiated points	2
Number of treatment sessions	1 session
Total radiant energy	18J
Application technique	contact
Operating mode	Continuous wave
Photosensitizer	methylene blue (0.005%)

The blood samples were collected from the aortic artery of the mice by exsanguination, for a cell count by using Sysmex^™^ C9.0 Software for the hemogram test. For an evaluation of lung inflammation in the BAL (bronchoalveolar lavage), the animals were tracheostomized and cannulated. Their lungs were washed with 3x0.5ml phosphate buffered saline (PBS). The volume of recovered BAL was centrifuged (1600rpm, 5 min at 4°C). The supernatant was collected and stored at -70°C for the cytokine analyzes by enzyme-linked immunosorbent assays (ELISA). The cell button was resuspended in 1 ml of PBS and was used for the total cell count. Ten microliters of the samples were added to Trypan Blue for a total cell counting with a Neubauer chamber. One hundred microliters were used to prepare the laminae for a differential counting of the cells (5 min, 1900rpm, 4°C) (Cytospin II—Shandon Instruments, Sewickley, PA, USA). The staining of the slides was performed with Instant-Prov. Three hundred cells were counted per laminae [52–55]. The lung fragments were fixed in a 4% solution of paraformaldehyde, with 0.1 M of Sorensen’s phosphate buffer, at pH 7.4, at 4°C, for 24 h in anhydrous alcohol, followed by dehydration in alcohol and by diaphanization in xylol. The fragments were embedded in paraffin, sectioned to 5 μm with the aid of a microtome (HYRAX M60, Zeiss, GR), de-paraffinized, cut to a thickness of 5 μm, and then stained with periodic acid-Schiff for analyzes of the mucus [55]. The protocol was detailed in S2 Fig. The internal and external limits of the respiratory epithelium was delimited (Image Pro-Plus 7.0) The mucus area was determined by area of glycoprotein component relation to total area of the respiratory epithelium. The results are expressed as the percentage (%). The measurements were performed in the five airways of each animal at ×400 magnification [60].

The hemi-jaws were dissected and fixed in 10% buffered formalin solution (Merck & Co. Inc, New Jersey, USA) at pH 7 for a period of 24–48 hours. They were decalcified in 4% EDTA for 2 months. The mandibles were then dehydrated in alcohol solutions and diaphanized in xylol. The fragments were embedded in paraffin, sectioned to 5 μm with a microtome (HYRAX M60, Zeiss, GR), de-paraffinized, cut to a thickness of 5 μm and stained with hematoxylin and eosin. Serial cuts were used involving the best specimens.

The morphometric analyzes were performed by a single examiner. Kappa values were used in order to measure the intra-observer agreement of measures. The observer was an experienced pathologist, França CM, [61–65] and he had an intra-observer agreement of 0.85. This pathologist followed some references [56–58] in order to proceed with the morphometric analyzes. The histological sections were photographed by using an Olympus Bx43 microscope with the help of Olympus cellSens^™^ software. The measurements of bone loss were performed with Image J Software Version 1.45. The distance between the cementum-enamel junction (CEJ) to the alveolar bone (the distal region of the first molar) was measured in millimeters. The linear measurements of each slide were summed in order to obtain an average value for each animal. The concentrations of cytokines were determined in the supernatant samples of the lavage fluid and the serum. The results were expressed as picograms of cytokine produced per mL. The Interleukin Th2 (IL-10, IL-4, IL-5, IFN-γ) and the Th1 (IL-1, IL-6, INF-γ) profiles were quantified by using ELISA (BioLegend, San Diego, USA). The determinations were performed in duplicate for each sample by using standard curves and following the manufacturer’s specifications. The quantification of serum alkaline phosphatase was also quantified by using ELISA (BioLegend, San Diego, USA).

Statistical analyzes were performed by a blind statistician using the GraphPad Prism program (GraphPad Software, Inc). The Kolmogorov-Smirnov test was used in order to determine the data distribution. Since the data was parametric, a one-way analysis of variance (ANOVA) was employed, followed by the Student-Newman-Keuls test. A p-value < 0.001 was considered indicative of statistical significance.

## Results

**Fig 2:**
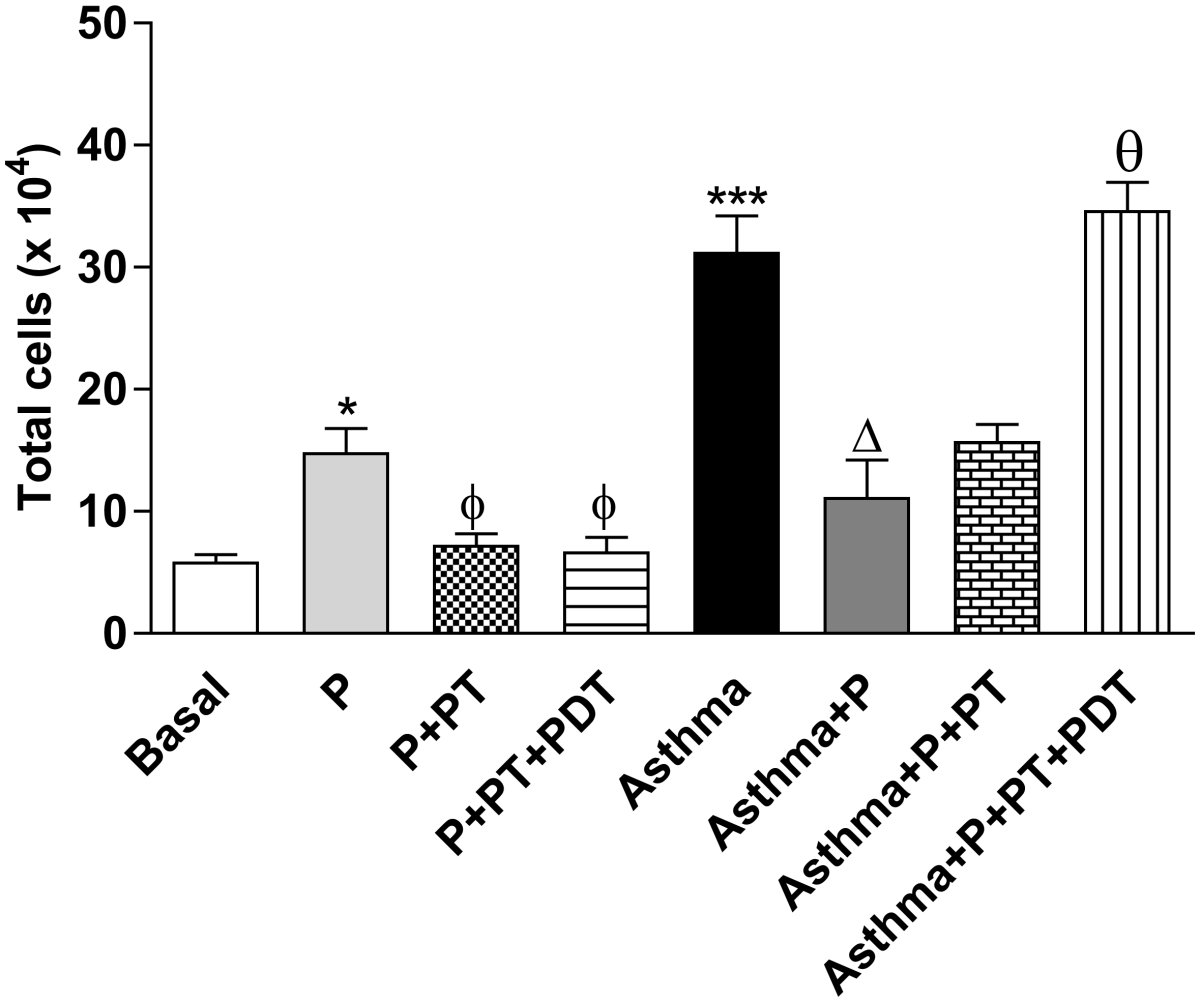
Quantification of the total number of cells that were recovered from the BAL. The volume of recovered BAL was used for the total cell count with Trypan Blue (x10^4^) with a Neubauer chamber. The groups were: Basal (unmanaged animals), P (periodontitis), P + TP (periodontitis + periodontal treatment), P + TP + PDT (periodontitis + periodontal treatment + photodynamic therapy), Asthma (asthmatic animals + periodontitis), Asthma + P + TP (asthmatic animals + periodontitis + periodontal treatment), Asthma + P + PT + PDT photodynamic therapy). The results are shown as mean ± SEM of the 2 experiments (n = 4–5). ANOVA was employed, followed by the Student-Newman-Keuls test. *p<0.05; *** p <0.001 when compared to the Basal group; ^ϕ^p <0.05 when compared to the P group; Δp <0.001 relative to the Asthma group; ^θ^p <0.001 when compared to the Asthma + P group.

The quantification of the cells that were recovered in the bronchoalveolar lavage (BAL) of the asthmatic mice (Fig 2) shows that the total count of the inflammatory cells (x10^4^/ml) in the bronchoalveolar lavage (BAL) was increased in the periodontitis (P) group (p <0.05) and in the asthma group (Asthma) (p <0.001) when compared with the basal group (B). The periodontal treatment (P + PT) (p <0.05) and the periodontal treatment associated with PDT (P + TP + PDT) (p <0.05) were able to decrease the total amount of inflammatory cells in the bronchoalveolar lavage when compared to the periodontitis (P) group. The association of the periodontitis with asthma (P + Asthma) reduced the number of the total cells that were recovered in the BAL when compared to the group with asthma (Asthma) (p <0.001). There were no differences in the total number of BAL cells after the standard periodontal treatments in the asthmatic mice (Asthma + P + TP) when compared with the asthmatic mice in the periodontitis group (Asthma + P). On the other hand, when photodynamic therapy was associated (Asthma + P + TP + PDT), there was a significant increase in the total number of cells (p <0.001) in relation to the periodontitis with asthma group (Asthma + P).

**Fig 3:**
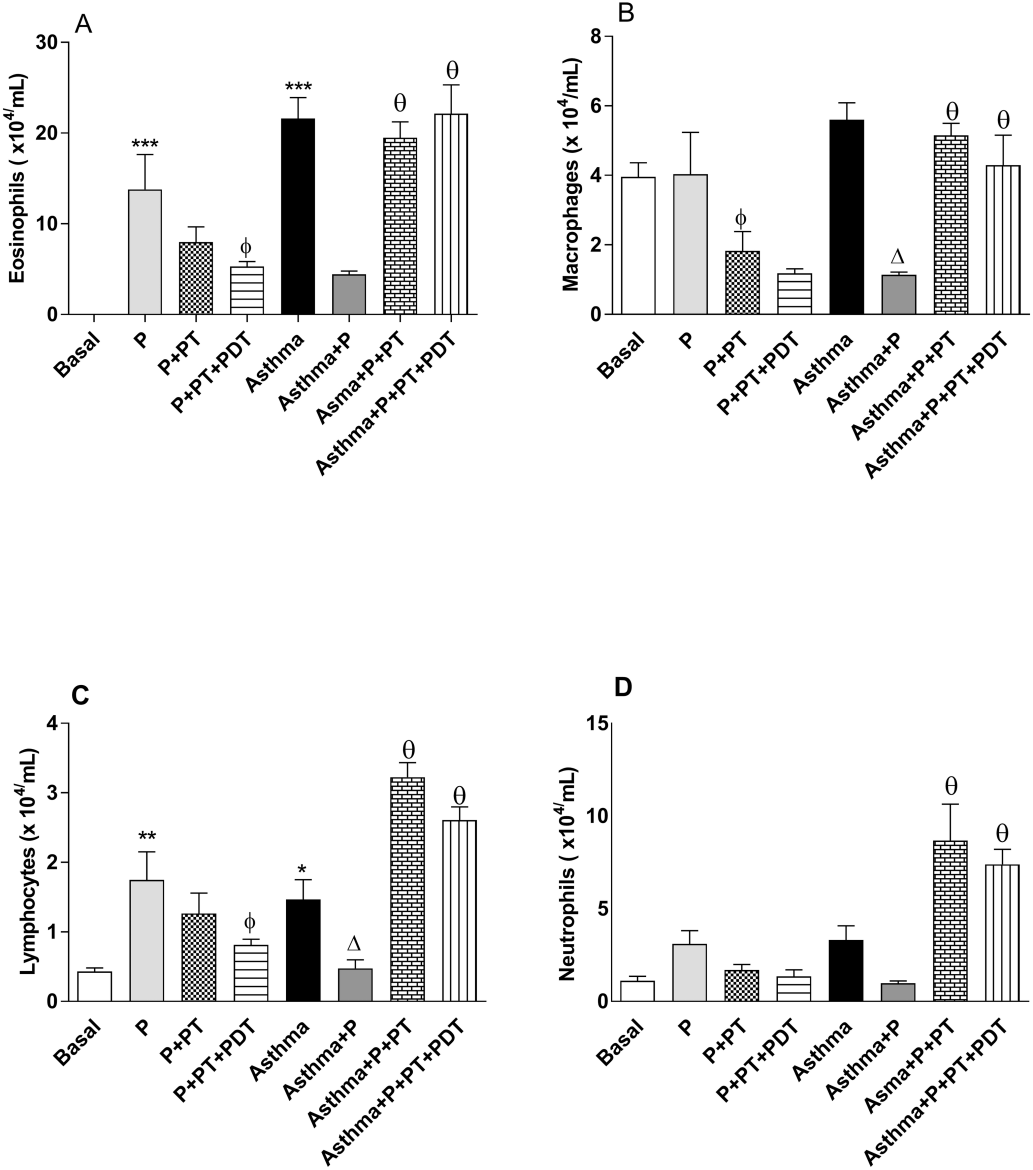
Quantification of the differential number of cells that were recovered from the BAL. BAL were used to prepare the stained slides (Instant-Prov) for a differential counting of the cells (x10^4^/mL). The groups were: Basal (unmanaged animals), P (periodontitis), P + PT (periodontitis + periodontal treatment), P + PT + PDT (periodontitis + periodontal treatment + photodynamic therapy), Asthma, Asthma + P (asthmatic animals + periodontitis), Asthma + P + PT (asthmatic animals + periodontitis + periodontal treatment), Asthma + P + PT + PDT (periodontitis + periodontal treatment + photodynamic therapy). The results are shown as mean ± SEM for the 2 experiments (n = 4–5). ANOVA was employed, followed by the Student-Newman-Keuls test. (A) φp <0.05 compared to the P group; Δp <0.001 relative to the Asthma group; Θp <0.01 compared to the Asthma + P group for the Asthma + P + PT group; Θp <0.01 in relation to the Asthma + P group in relation to the Asthma + P + PT + PDT group. (B) ** p <0.001, * p <0.01 compared to the Basal group; Φp <0.001 compared to the P group; Δp <0.05 compared to the Asthma group, θp <0.001 compared to the Asthma + P group for both groups. (C) θp <0.001 compared to the Asthma + P group for both groups. (D) *** p <0.001 compared to the baseline; Φp <0.001 compared to the P group; Θp <0.001 compared to the Asthma + P group for the Asthma + P + PT group; Θp <0.001 compared to the Asthma + P group for the Asthma + P + PT + PDT group.

In the quantification of the differential number of cells that were recovered from the BAL (Fig 3) there was an increase (p<0.001) in eosinophils (Fig 3A) for the (P) and (Asthma) groups in relation to the Basal group. The treatment with PDT decreased (p <0.001) the amount of eosinophils in the (P + TP + PDT) group when compared to the (P) group. When associated with asthma, the two modalities of TP (Asthma + P + TP) and (Asthma + P + TP + PDT) increased (p<0.001) the number of eosinophils when compared with the (Asthma + P) group. There was a reduction (p<0.05) in the macrophage differential count (x10^4^/ml) of the BAL for the (P+PT) group in relation to the (P) group. When we associated asthma with P (Asthma + P), there was a reduction of cells (p<0.001) in relation to the Asthma group. The periodontal treatment (Asthma + P + TP) and PDT (Asthma + P + TP + PDT) in the asthmatic mice increased (p<0.001) the amount of macrophages when compared to the (Asthma + P) group. The amount of lymphocytes (Fig 3B) increased (p<0.001) for the (P) group and for the (Asthma) group (p <0.01) when compared to the (Basal) group. For the (P+PDT) group, there was a decrease (p <0.001) in relation to the (P) group. For the (Asthma + P) group, there was a decrease (p <0.05) in relation to the (Asthma) group. When the treatments were associated, the (Asthma + P + TP) group and the (Asthma + P + TP + PDT) group had increased (p <0.001) lymphocytes in relation to the (Asthma + P) group. There was an increase in neutrophils (p<0.001) (Fig 3C) for the (Asthma + P + TP) and (Asthma + P + TP + PDT) groups when compared to the (Asthma + P) group.

**Fig 4:**
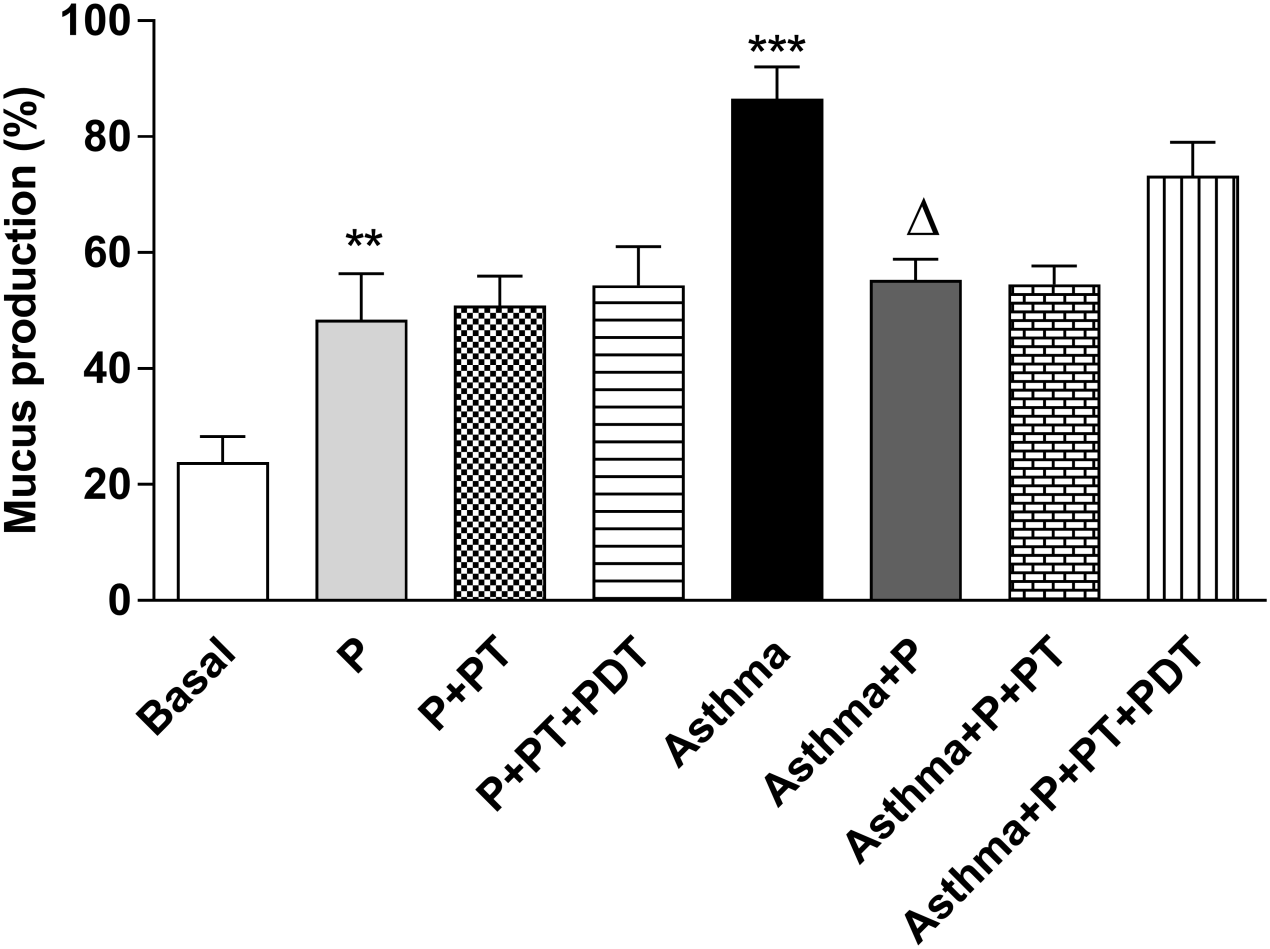
Quantification of mucus in the airways. For evaluation of mucus production, lung morphology analysis were realized with Periodic acid-Schiff histochemical (PAS) to characterize the glycoprotein component of Goblet cells. Five-micrometer-thick sections were analyzed for mucus deposition quantification as described in S2 Fig. The results are expressed as the percentage (%). The groups were: Basal (unmanaged animals), P (periodontitis), P + PT (periodontitis + periodontal treatment), P + PT + PDT (periodontitis + periodontal treatment+ photodynamic therapy), Asthma, Asthma + P (asthmatic animals + periodontitis), Asthma + P + PT (asthmatic animals + periodontitis + periodontal treatment), Asthma + P + PT + PDT + Asthma (periodontitis + periodontal treatment + photodynamic therapy). The results are shown as mean ± SEM for the 2 experiments (n = 4–5). ANOVA was employed, followed by the Student-Newman-Keuls test. ** p <0.01; *** p <0.001 when compared to the basal group; Δp <0.001 relative to the Asthma group.

**Fig 5:**
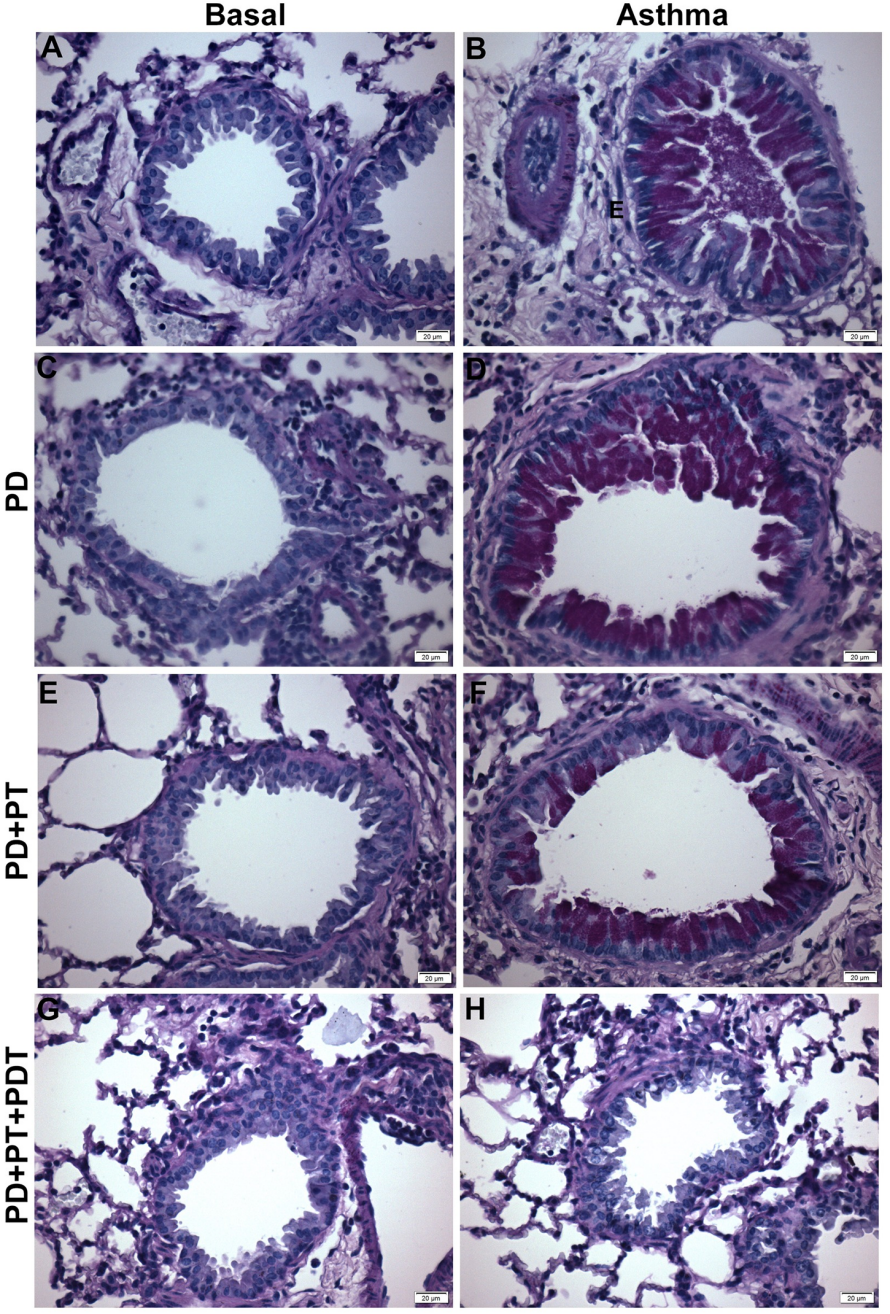
Reduced mucus secretion in the lungs of periodontitis induced animals. Asthmatic animals were submitted to different protocols as described in the material and methods. Further all animals were euthanized and lungs were sectioned and stained with PAS—Periodic Acid Schiff (400x) as described in S2 Fig. Different groups can be observed (A-H): A- Basal, B- Asthma (A), C- Periodontitis (P), D- Induction of Asthma + Induction of Periodontitis (Asthma+PD), E- Induction of Periodontitis + Standard Periodontal Treatment (P+TP), F- Induction of Asthma + Induction of Periodontitis + Standard Periodontal Treatment (Asthma+P+TP), G- Induction of Periodontitis + Standard Periodontal Treatment + Photodynamic Therapy (P+PT+PDT), H-Asthma Induction + Induction of Periodontitis + Standard Periodontal Treatment + Photodynamic Therapy (Asthma+P+TP+PDT). Data representative of 2 experiments. (n = 4–5 animals per group). Bar = 20 μm.

For the quantification of mucus in the airway (Figs 4 and 5) there was an increase (p<0.001) in the mucus production in the asthma (A) and periodontitis (P) groups (p <0.01) when compared to the basal (B) group. The association of P with asthma (Asthma + P) decreased the mucus production when it was compared to the (Asthma) group.

**Fig 6:**
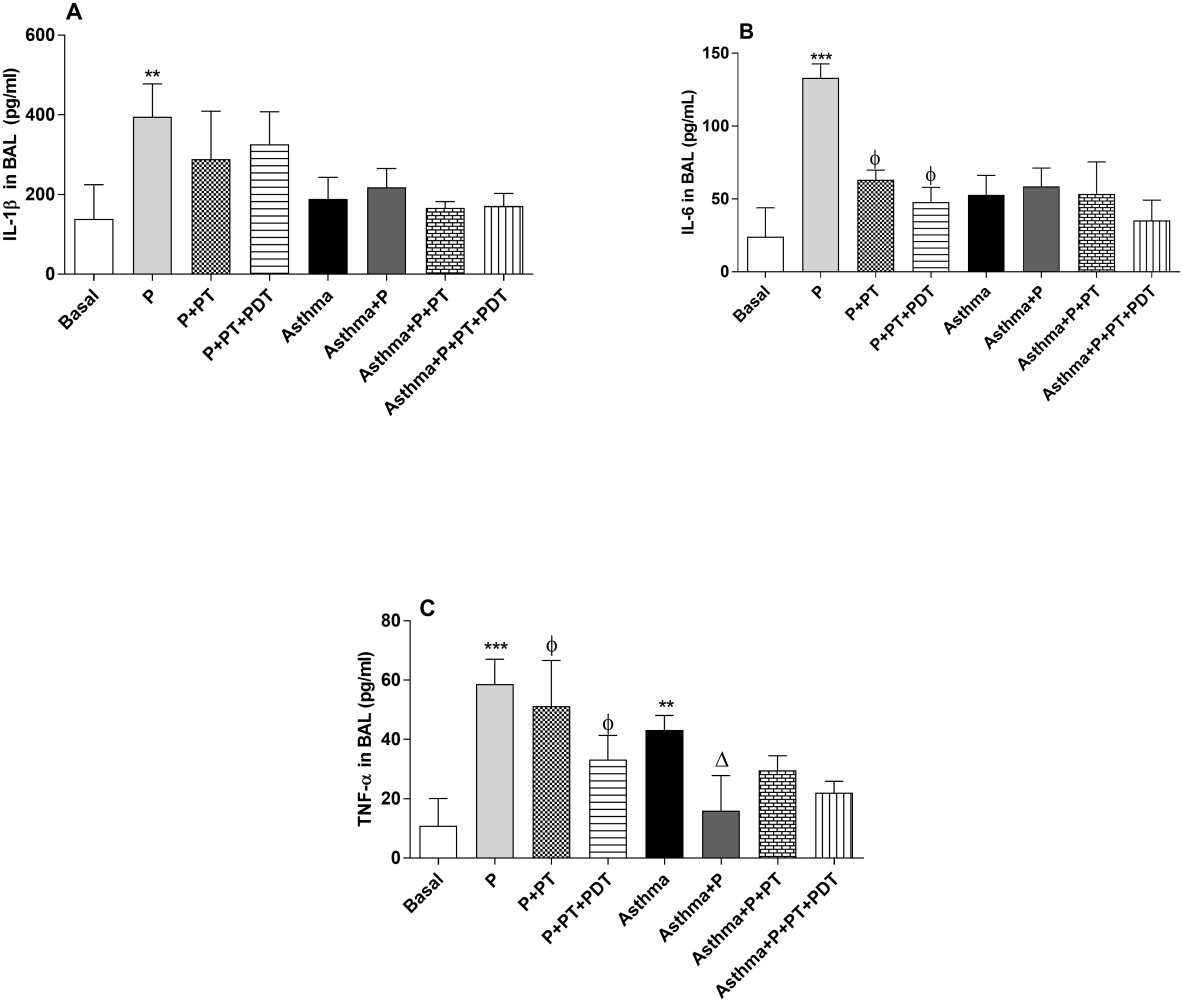
Analyzes of the cytokines IL-1β (A), IL-6 (B) and TNF-α (C) in the BAL. For an evaluation of lung inflammation in the BAL the lungs were washed with phosphate buffered saline and after centrifugation, the supernatant was collected for the cytokine analyzes by ELISA. The groups were: Basal (unmanaged animals), P (periodontitis), P + PT (periodontitis + periodontal treatment), P + PT + PDT (periodontitis + periodontal treatment + photodynamic therapy), Asthma, Asthma + P (asthmatic animals + periodontitis), Asthma + P + PT (asthmatic animals + periodontitis + periodontal treatment), Asthma + P + PT + PDT + Asthma (periodontitis + periodontal treatment + photodynamic therapy). The results are shown as mean ± SEM for the 2 experiments (n = 4–5); ANOVA was employed, followed by the Student-Newman-Keuls test. (A) ** p <0.01 related to the Basal group; (B); *** p <0.001 when compared to the basal group; Φ p <0.001 in relation to the P group; (C) ** p <0.05; *** p <0.001 when compared to the basal group; Φp <0.05 for the group P + TP when compared to the P group and φ p <0.01 for the group P +TP + PDT when compared to the P group; Δp <0.05 when compared to the Asthma group.

For the analyzes of the IL-1β, IL-6 and TNF-α cytokines in the BAL supernatant, the inflammatory cytokines (Fig 6) related to the Th1 mechanism were analyzed. An increase (Fig 6A) (p <0.01) of IL-1β production in the periodontitis (P) group was observed when compared to the basal group (B). There were no differences in the other groups that were analyzed (p> 0.01). There was an increase (p<0.001) in the IL-6 levels in the BAL of the (P) group when compared to the basal (B) group (Fig 6B). On the other hand, there was a reduction (p<0.001) in the IL-6 production in the treatment groups (P + TP) and (P + TP + PDT) when compared to the (P) group. We also observed an increase (p<0.001) in the production of TNF-α (Fig 6C) for the (P) and (A) groups when compared with the basal (B) group. It was observed that the standard periodontal treatment (P+TP) alone or in a combination with the photodynamic therapy (P + TP + PDT) decreased the production of TNF-α in the BAL, respectively (p<0.001 and p<0.01), when compared with the (P) group. The association of P with asthma (Asthma + P) decreased (p <0.05) the TNF-α production when compared to its control group, the (Asthma) group.

**Fig 7:**
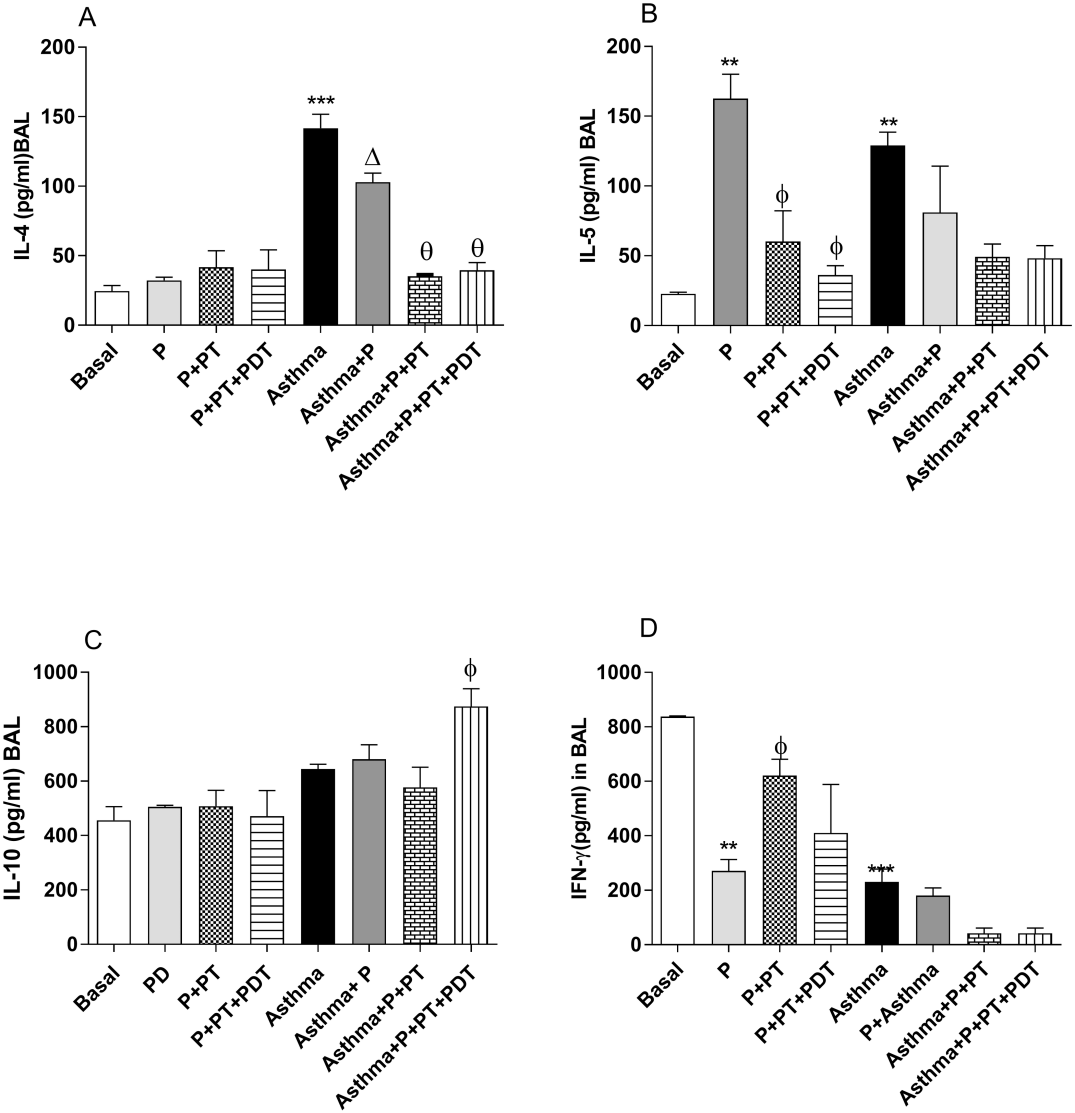
Analyzes of the IL-4 (A), IL-5 (B), IL-10 (C) and INF-γ (C) cytokines in the BAL. For an evaluation of lung inflammation in the BAL the lungs were washed with phosphate buffered saline and after centrifugation, the supernatant was collected for the cytokine analyzes by ELISA. The groups were: Basal (unmanaged animals), P (periodontitis), P + PT (periodontitis + periodontal treatment), P + PT + PDT (periodontitis + periodontal treatment + photodynamic therapy), Asthma, Asthma + P (asthmatic animals + periodontitis), Asthma + P + PT (asthmatic animals + periodontitis + periodontal treatment), Asthma + P + PT + PDT + Asthma (periodontitis + periodontal treatment+ photodynamic therapy). The results are shown as mean ± SEM for the 2 experiments (n = 4–5); ANOVA was employed, followed by the Student-Newman-Keuls test. (A) *** p <0.001 relative to the Basal group; Δp <0.001 relative to the Asthma group; Θp <0.001 compared to the Asthma + P group compared to the Asthma + P + PT group and θp <0.01 compared to the Asthma + P group compared to the Asthma + P + PT + PDT group; (B) ** p <0.01 compared to the basal group; φ <0.01 compared to the P group compared with the P + PT group, and φ <0.001 compared to the P group compared with the P + PT + PDT group. (C) φ p <0.05 in relation to the P group; (D) * p <0.05 *** p <0.001 compared to the Basal group; Φ 0.001 in relation to the P group.

For the analyzes of the IL-4, IL-5 IL-10 and IFN-γ cytokines in the BAL supernatant–the cytokines related to the mechanism (Th2) of asthma (Fig 7) were evaluated in the BAL supernatant. There was an increase (p <0.001) in the production of IL-4 in the (A) group when compared to the basal (B) group (Fig 7A). The association of P and asthma (P + A) decreased (p<0.001) the production of IL-4 in relation to the (Asthma) group. In addition, the periodontal treatment (Asthma+P+TP) and the periodontal treatment with PDT in the asthmatic mice with P (Asthma+P+TP+PDT) decreased (p<0.01) the level of IL-4 in the BAL when compared to the (Asthma+P) group. There was an increase (p<0.01) in the IL-5 levels (Fig 7B) of the BAL in the (P) and (Asthma) groups when compared with the basal (B) group. On the other hand, there was a decrease (p<0.01) in IL-5 when the periodontal treatments of (P+TP) and PDT were performed, (P+TP+PDT) when compared to the (P) group. It was observed (Fig 7C) that the PDT treatment (A+P+TP+PDT) in asthmatic mice increases the IL-10 production in the BAL when compared to the Asthma + P group (A+P). A decrease in the IFN-y levels (p<0.05) in the periodontitis (P) and asthma (Asthma) groups when compared to basal (B) group was observed in Fig 7D. The standard periodontal treatment (P+TP) increases IFN-γ.

**Fig 8:**
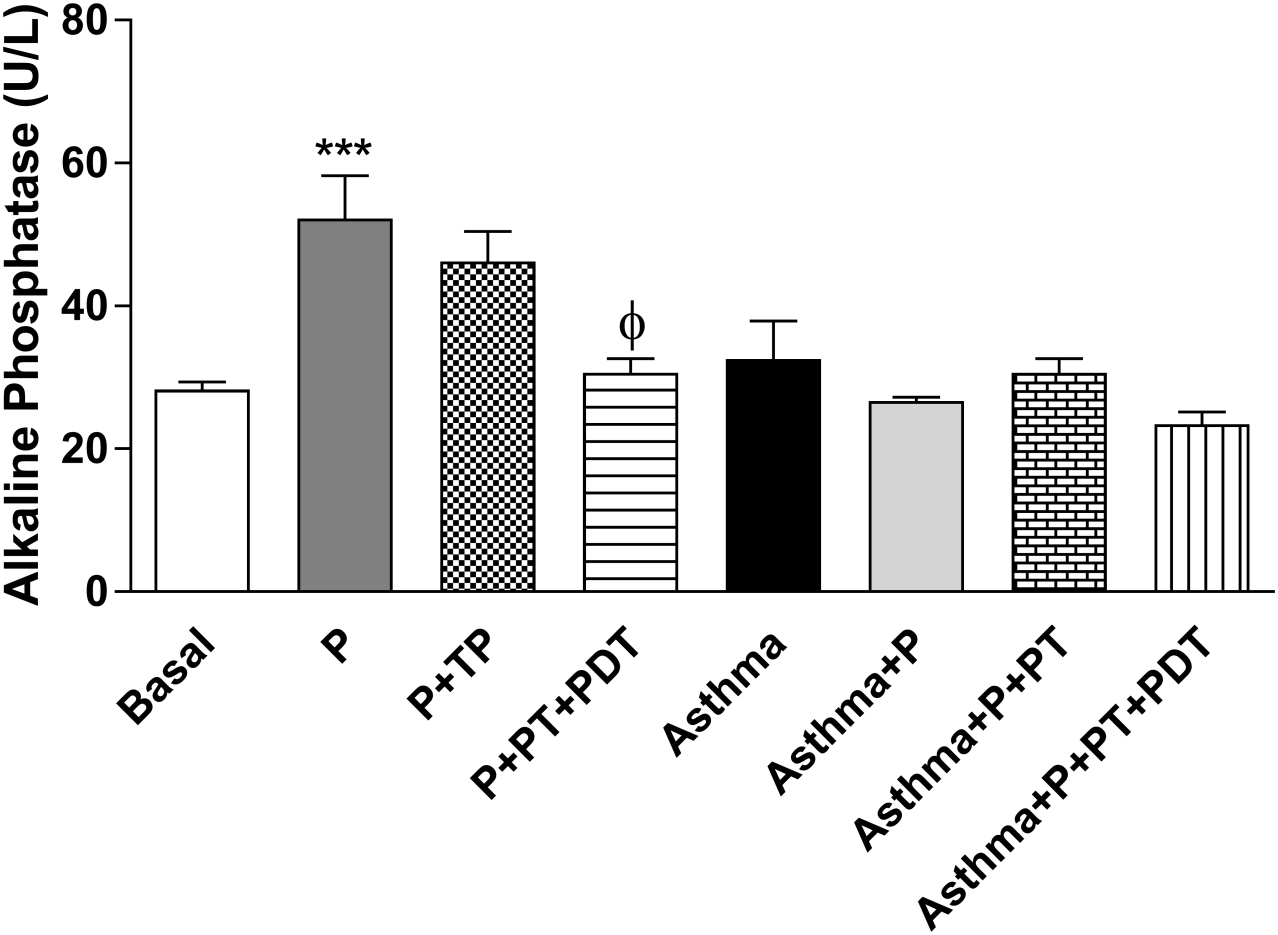
Quantification of alkaline phosphatase in the serum. The quantification of serum alkaline phosphatase was also quantified by using ELISA. The groups were: Basal (unmanaged animals), P (periodontitis), P + PT (periodontitis + periodontal treatment), P + PT + PDT (periodontitis + periodontal treatment + photodynamic therapy), Asthma, Asthma + P (asthmatic animals + periodontitis), Asthma + P + PT (asthmatic animals + periodontitis + periodontal treatment), Asthma + P + PT + PDT + Asthma (periodontitis + periodontal treatment+ photodynamic therapy). The results are shown as mean ± SEM for the 2 experiments (n = 4–5); ANOVA was employed, followed by the Student-Newman-Keuls test. *** p <0.001 compared to the Basal group; Φp <0.01 compared to the P group.

Quantification of serum alkaline phosphatase—there was an increase in the production of alkaline phosphatase (U/L) (Fig 8) in the (P) group when compared to the basal (B) group (p<0.001). PT that was associated with PDT (P+ PT+PDT) decreased the alkaline phosphatase production (p<0.01) in relation to the (P) group.

**Fig 9:**
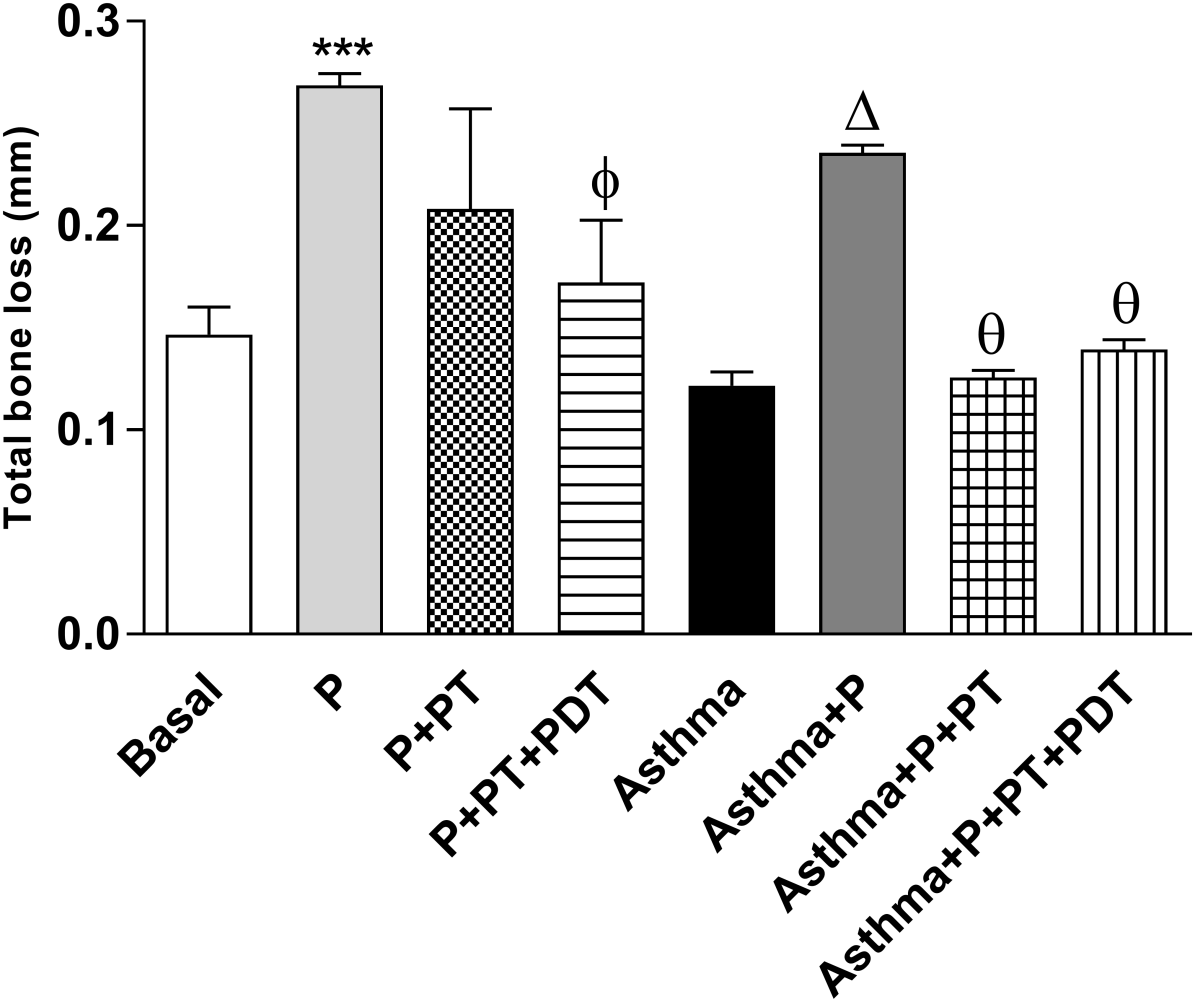
The distance between the cementum-enamel junction (CEJ) and the alveolar bone measure in millimeters (mm). The distance between the cementum-enamel junction (CEJ) and the alveolar bone (the distal region of the first molar) was measured in millimeters (mm). The linear measurements of each slide were summed in order to obtain an average and ±sd value. The histological slides were stained with hematoxylin and eosin solution (256x). The groups were: Basal (unmanaged animals), P (periodontitis), P + PT (periodontitis + periodontal treatment), P + PT + PDT (periodontitis + periodontal treatment + photodynamic therapy), Asthma, Asthma + P (asthmatic animals + periodontitis), Asthma + P + PT (asthmatic animals + periodontitis + periodontal treatment), Asthma + P + PT + PDT + Asthma (periodontitis + periodontal treatment+ photodynamic therapy). The results are shown as mean ± SEM for the 2 experiments (n = 4–5); ANOVA was employed, followed by the Student-Newman-Keuls test. *** p <0.001 when compared to the basal group; Φp <0.01 when compared to the P group; Δp <0.001 related to the Asthma group; Θ <0.001 when compared to the Asthma + P group and θ <0.01 related to the Asthma + P group when compared to the Asthma + P + PT group.

**Fig 10:**
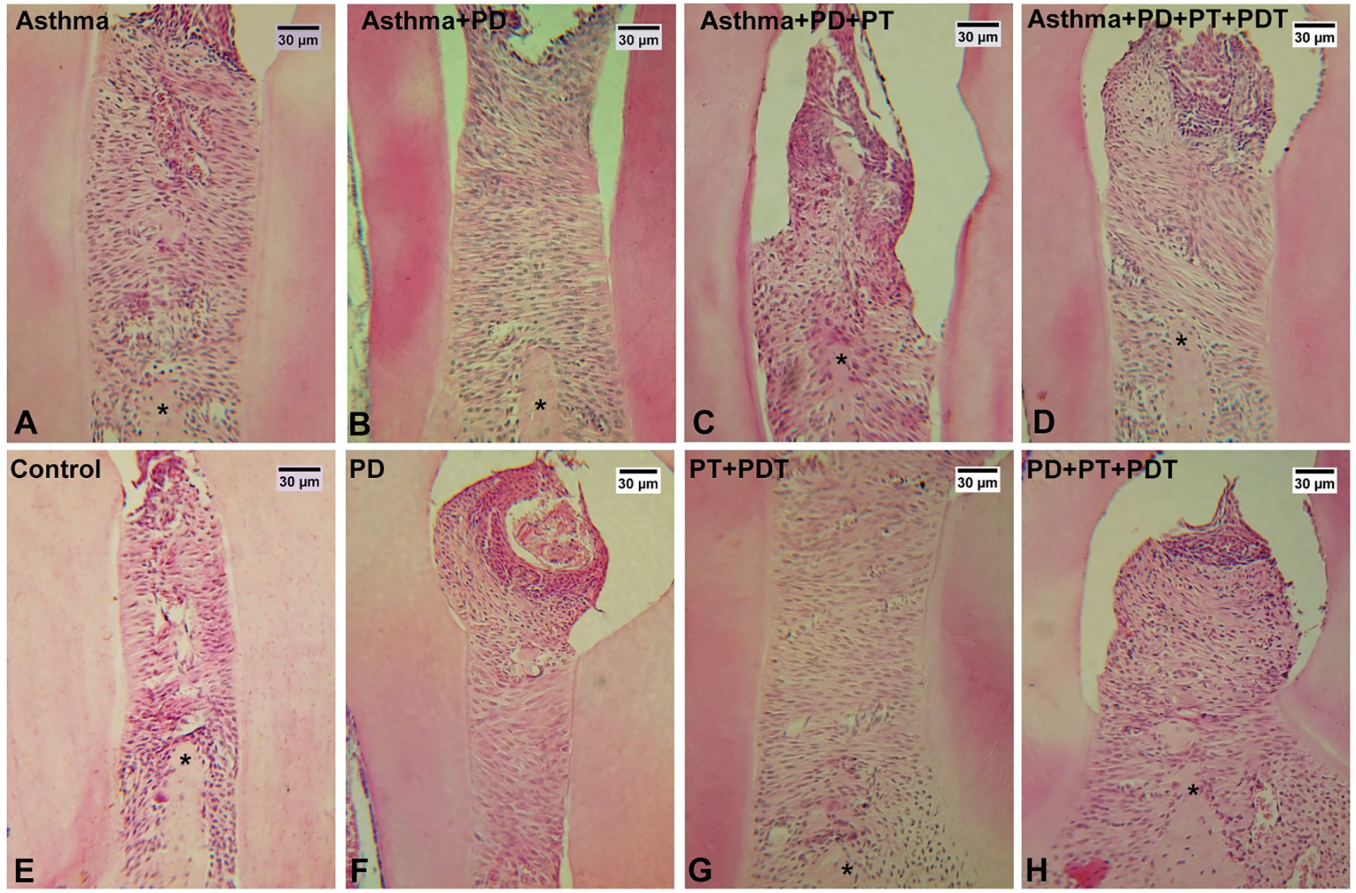
Histological analysis of alveolar bone between the 1st and the 2nd molar. (A-H): A- Basal, B- Periodontitis (P), C- P+PT (Periodontal treatment), D- P+PT+PDT (photodynamic therapy, E- Asthma (A), F- A+P, G- A+P+PT, and H -A+P+PT+PDT. Decalcified periodontal mandibles were sectioned (5μm). Decalcified periodontal tissue sections (256x) were stained with HE (R HEhematoxylin and eosin solution) Representative tissue sections are shown. (*) represents alveolar bone. Bar = 30 μm.

An increase (p<0.001) in bone resorption was observed in the (P) group when compared to the basal (B) group. (Table 2) PT that was associated with PDT (P+PT+PDT) was able to decrease the bone resorption (p<0.01) in the alveolar bone region when compared to the (P) group. The asthmatic mice in which P was induced (Asthma+P) presented a greater resorption (p<0.001) than did the group in which only asthma (A) was induced. The PT (Asthma+P+PT) and PDT (Asthma+P+PT+PDT) groups had lower values of bone resorption (p<0.01) than did the asthmatic mice with periodontitis (Asthma+P). These values are shown in Fig 9 and illustrated in Fig 10.

**Table 2:** Total bone loss.

Total bone loss (mm)								
Group	Basal	P	P+PT	P+PT+PT	Asthma	Asthma+P	Asthma+P+PT	Asthma+P+PT+PDT
**Mean**	0.117	0.263	0.205	0.182	0.121	0.235	0.125	0.139
**±sd**	0,002	0,005	0,049	0,049	0,011	0,006	0,006	0,008

sd- standard deviation, P- Periodontitis group, PT-Periodontal treatment, PDT-Photodynamic therapy,

## Discussion

Periodontitis is defined as a chronic inflammation that destroys the supporting tissues of the teeth [66]. It was characterized based on high levels of bone resorption [56–58], and alkaline phosphatase [67] which are well-established methods in the literature. As we described before, periodontitis is characterized by an acute inflammatory process, which is then characterized by a pro-inflammatory profile of cytokine release, known as Th1 [3]. Among the cytokines with a Th1 profile, we can cite IL-1β, IL-6 and TNF-α [5,6,7]. Periodontitis group (P) increased the amount of alkaline phosphatase and bone resorption, thus validating the experimental model of periodontitis and increase all the Th1 cytokines profile.

The asthma model involved the induction of an allergic reaction using ovalbumin, which is recognized as mimicking signs and symptoms found in asthmatic patients [40–43]. The experimental model for the induction of asthma has increased cellularity in the BAL represented by eosinophils, lymphocytes and neutrophils, as well as an increase in cytokines with a Th2 profile (IL-4 and IL-5), together with the production of mucus in the airways. This was an expected result for the asthmatic animals. To better understand the mechanism linking these two diseases, we studied the Th1/Th2 immunoregulatory control through its different cytokine profiles [9].

Interestingly, the combination of asthma and periodontitis led to a significant reduction in the inflammatory cells (eosinophils, lymphocytes, macrophages) as well as the IL-4 cytokines release in the lungs. This decrease was accompanied by a reduction in the amount of mucus. It can therefore be inferred that periodontitis exerted some influence on pulmonary inflammation. In this group (A+P), bone resorption remained high, showing that periodontitis was still active. These findings are in agreement with data described previously by researchers studying the association between periodontitis and allergy [21]. This study revealed an inverse relationship between periodontitis and allergy in a sample of 2.837 individuals. The authors discussed whether exposure to periodopathogens may influence the asthma progression. In other words, the regulation of the Th1-Th2 balance was deviated to the Th1 response in the asthmatic mice when periodontitis was associated with asthma. For these reasons, we have inferred that our results have an inverse relationship [19–24].

Conversely, in a recent study with 5.976 patients, a positive association was found between periodontitis and asthma [11], while there is an inverse association only when the patients have been taking antiasthmatic medication [11]. The causal relationship between them is still unclear [30]. Further studies are needed to elucidate the link between these two pathologies [19–29].

Considering the controversial data regarding the correlation between periodontitis and asthma, we have evaluated the effects of its treatment with conventional periodontal treatment and with PDT.

When comparing the effectiveness of the two periodontal treatments in the mice without asthma, we noticed that the conventional treatment decreased the macrophages, as well as IL-6 and TNF-α, corroborating with the literature [3–7]. However, it was interesting to note that the treatments appeared to act slightly differently, since PDT decreased the number of eosinophils and IL-5 in the mice without asthma and caused less bone resorption as well. Further studies are necessary to better understand the influence od PDT in Th2 mechanisms.

In the asthmatic mice, after conventional periodontitis treatment, an increase in macrophages, lymphocytes, neutrophils and eosinophils were found. This variation in cellularity was followed by decreased IL-4 and TNF-α production. Moreover, standard treatment was able to interrupt the bone resorption process, confirming what occurs in clinical practice. Interestingly, PDT in asthmatic mice also increased the total cell count (macrophages, lymphocytes, neutrophils and eosinophils) in the BAL. Although the increase in defense cells is a disadvantage of this form of treatment, the cytokine release pattern was favorable, as the anti-inflammatory cytokine IL-10 was released in a greater quantity only for this group. These results suggest that PDT could stimulate the cellular immune response as demonstrated by some authors [68]. Further studies could investigate if both M2 (macrophages) and Treg (regulatory T cells) are implicated in these processes [69] producing anti-inflammatory cytokines [70,71].

Although the mechanisms of the action are different, when photobiomodulation was directly applied in the lungs, has reduced the TNF-α expression after an acute immunocomplex lung injury in rats [72]. They tested different doses of laser and they found a dose-dependent reduction of TNF-α levels in acute inflammation [72]. In our study we also found a decrease in TNF-α levels in the BAL after a PDT. One limitation of this study was not to study the systemic alterations (serum) of cytokines release. More studies are needed in order to understand these systemic interactions.

Photodynamic Therapy is known as the first order of antimicrobial action in a periodontal treatment and has been largely studied [35,36,28,39,44–46]. Photosensitizers affect bacterial cells, damaging structures and impeding essential functions for bacterial survival [37]. We selected methylene blue because it is a widely studied photosensitizer for antimicrobial PDT, due to its absorption around 650nm and its photophysical and photochemical properties [36,39]. Besides that, it has a positive charge compound which binds to the bacteria due to an electrostatic interaction. Some studies have also shown an effective antibacterial action [44–47]. Methylene blue is commercialized by different companies and it may be used in different formulations/concentrations. Some well-known brands are HELBO^®^ and Chimiolux. In Brazil, we have selected methylene blue because is approved by the Brazilian Health Regulatory Agency (ANVISA), and is widely used for periodontitis treatment [44–47]. Besides, different chemical names for this molecule are also used, such as 3,7-Bis(dimethylamino)phenazathioniumchloride, Basic Blue 9, Tetramethyl Thionine Chloride and Phenothiazine Chloride. The nomenclature “phenothiazine” is not the most suitable of products since phenothiazines are tricyclic yellow compounds and not photosensitizers.

Finally, it was interesting to note that we had an unexpected result in one of our control groups (P+PT). Our data has shown that periodontitis group (P) *per se* caused an increase in the total number of cells, mainly in the eosinophils and lymphocytes, as well as in the IL-5 cytokine release and mucus production. Thus, this work has contributed in elucidating, in part, the relationship between P and asthma. Additional studies involving functional measurements of airway hyperreactivity and the investigation of structural changes in the airways are needed to gain a better understanding of the effects of periodontal treatment in patients with asthma.

In conclusion, periodontitis in the asthmatic mice reduced the inflammatory migrated cells in the BAL (eosinophils, lymphocytes, macrophages), as well as in reduce the levels of the IL-4 and TNF-α cytokines, which was additionally accompanied by a decreased mucus production. After the removal of the causative agent of the periodontal inflammation/infection (PDT treatment), the total cell counts increased, but this increase was not accompanied by a pro-inflammatory cytokines release. Only in the PDT group an anti-inflammatory cytokine (IL-10) was increased. More studies are needed in order to understand these mechanisms of action.

## Supporting information

S1 Fig Protocol for ligature induced in the lower first molar mice. (DOCX)

S2 Fig Protocol for lung morphology analysis for evaluation of mucus production. (DOCX)
