# Supplementary material for: Effects of periodontitis on the development of asthma: The role of photodynamic therapy
Source: PLoS One. 2017 Nov 16;12(11):e0187945. doi: 10.1371/journal.pone.0187945 (PMC5689838; doi:10.1371/journal.pone.0187945)
Supplement: S1 Fig — (DOCX) [file pone.0187945.s001.docx]

**Protocol for ligature induced in the lower first molar mice**

- The animals were anesthetized intraperitoneally with Ketamine 100mg / kg and Xilazine 10mg / kg (Ceva^®^, São Paulo, Brazil).
- The mice' mouth were mainteined oppen as follow: a cotton thread # 5 supported by two metal supports involved the palatal upper central incisors and the mandibule was supported by flat support.
- The tongue was withdraw with a insertion spatula developed for the research.
- The interproximal space between the first and second molars was gently inspected with a individualized K-type file (size # 08).
- A 6-0 ophthalmologic needle thread of nylon (Shalon^®^, São Paulo, Brazil monofilament black) was used. for the ligation technique
- The final 10 cm of this tread were cutted, and the needle discarded.
- This piece (10cm) was seized by two curved ophthalmic port needles developed for the study (Dlmicof^®^ - Derf model, Sao Paulo, Brazil) then gently inserted between the first and second right lower molar, into the interproximal space around the first molar.
- A simple node was performed twice over the vestibular face of the lower first molar.
