# Supplementary material for: Effects of periodontitis on the development of asthma: The role of photodynamic therapy
Source: PLoS One. 2017 Nov 16;12(11):e0187945. doi: 10.1371/journal.pone.0187945 (PMC5689838; doi:10.1371/journal.pone.0187945)
Supplement: S2 Fig — (DOCX) [file pone.0187945.s002.docx]

**Protocol for Lung Morphology Analysis for evaluation of mucus production**

- This procedure was performed after collection of BAL
- The lungs were removed and fixed in formaldehyde solution (10%), then processed, and embedded in paraffin
- Five-micrometer-thick sections were stained with Periodic acid-Schiff histochemical (PAS)
- PAS was used to characterize the glycoprotein component, of Goblet cells
- Slides were observed under a light microscope (Olympus Bx43- Tokyo, Japan)
- Photographed using the Cellscens Standard program, Tokyo, Japan)

**Mucus Deposition Quantification**

- The internal and external limits of the respiratory epithelium (that includes goblet cells) was delimited (Image Pro-Plus 7.0)
- The mucus area was determined by area of glycoprotein component of goblet cells in relation to total area of the respiratory epithelium
- The results are expressed as the percentage (%)
- A measure of similar. diameters were standardized to rule
